# Supplementary material for: Gamebird responses to anthropogenic forest fragmentation and degradation in a southern Amazonian landscape
Source: PeerJ. 2017 Jun 7;5:e3442. doi: 10.7717/peerj.3442 (PMC5466001; doi:10.7717/peerj.3442)

**Gamebird responses to anthropogenic forest fragmentation and degradation in a southern Amazonian landscape**

**Fernanda Michalski, Carlos A. Peres**

Supplementary Material

Figure S1: Land-use maps representing the evolution of the landscape structure over a 12-year period in the Alta Floresta region of southern Brazilian Amazonia from (A) 2004 to (B) 2016. Land-cover classes are represented by forest (green), non-forest (white) and water (blue). Total forest cover in this study region declined from 42% in 2004 to 35% in 2016. This 7,295 km^2^ study landscape is bounded by the Teles Pires river to the north and east. Forest cover was not substantially reduced over this period, but the level of forest degradation apparently increased substantially.


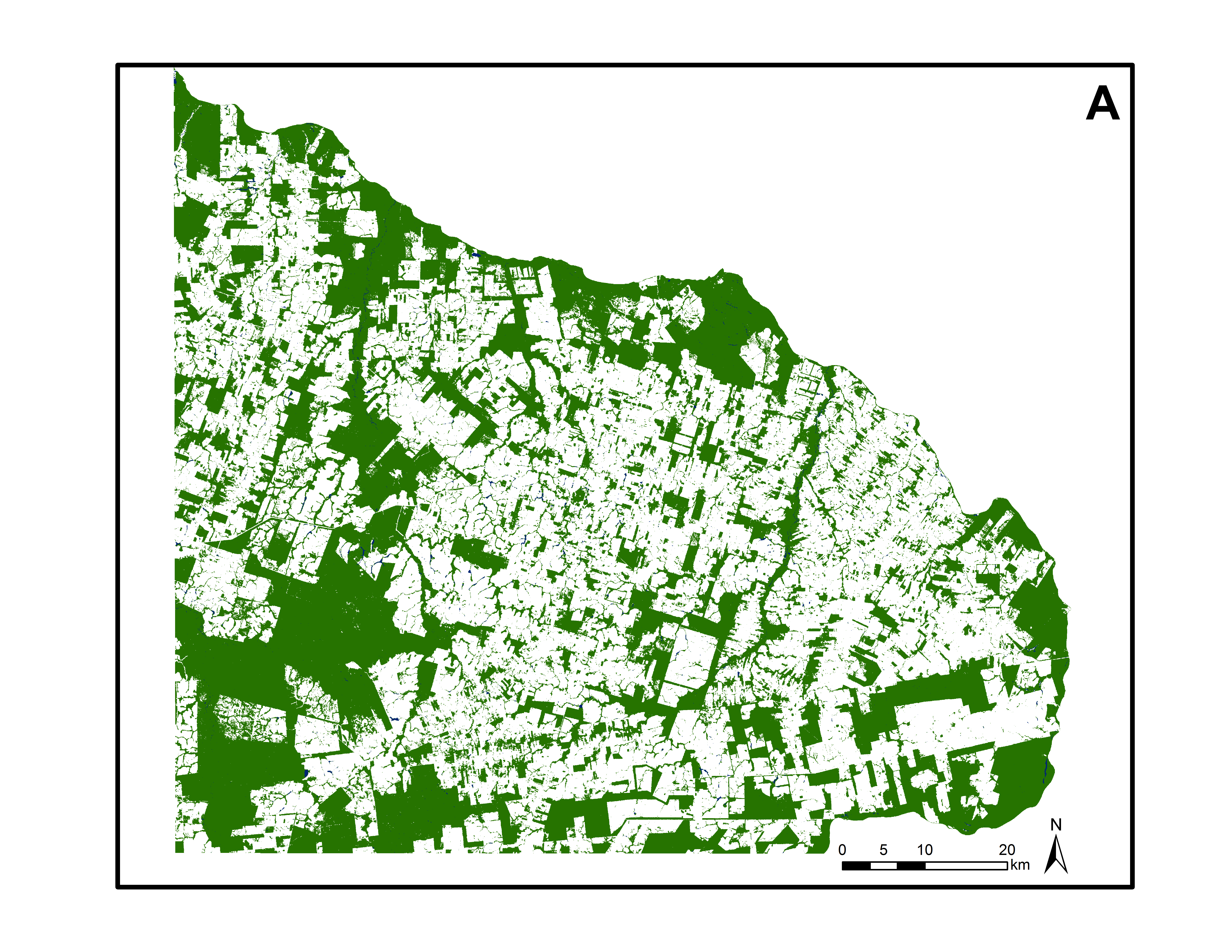


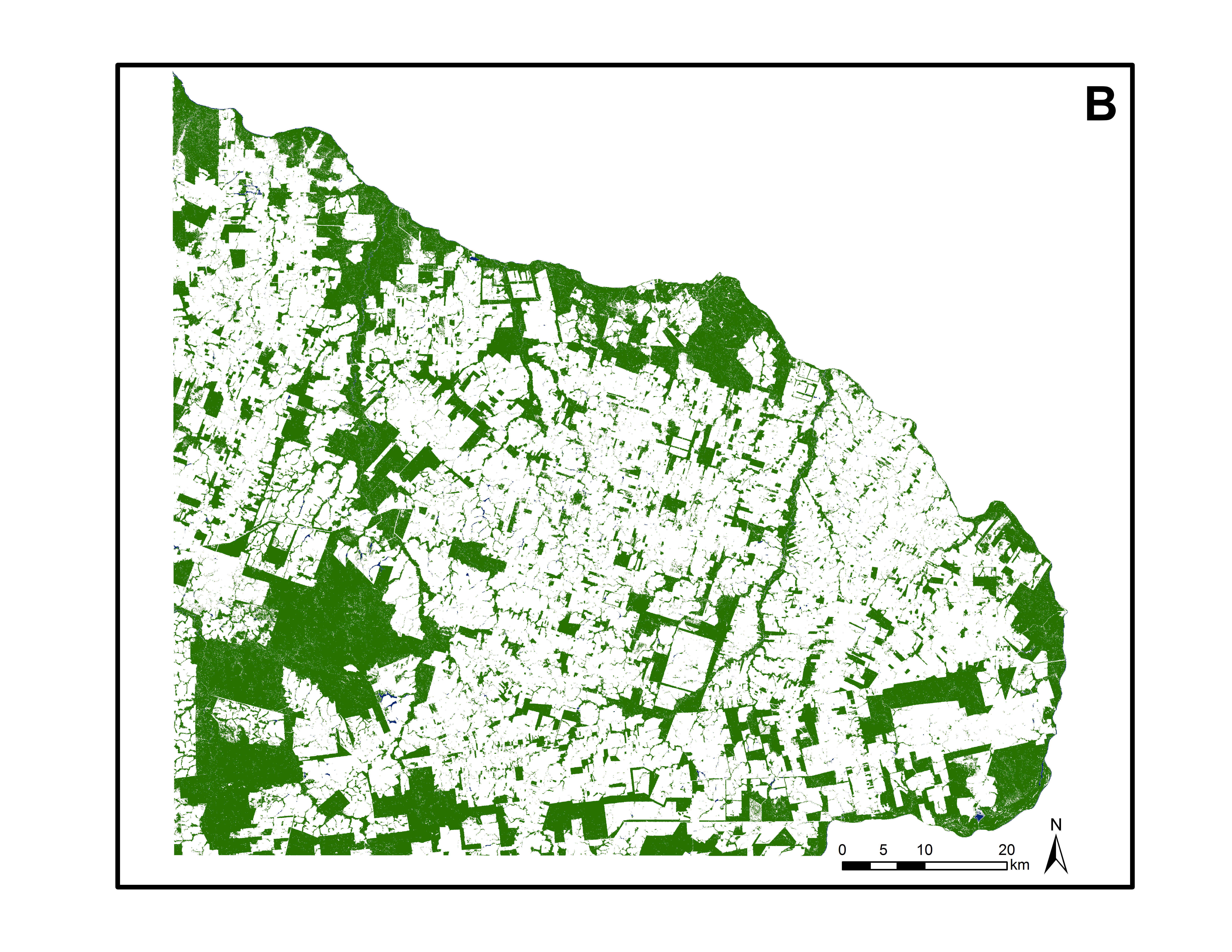

Supplement: Figure S1 — Land-cover classes are represented by forest (green), non-forest (white) and water (blue). Total forest cover in this study region declined from 42% in 2004 to 35% in 2016. This 7,295 km2 study landscape is bounded by the Teles Pires river to the north and east. Forest cover was not substantially reduced over this period, but the level of forest degradation apparently increased substantially. [file peerj-05-3442-s003.docx]
